# Hematoma block or procedural sedation and analgesia, which is the most effective method of anesthesia in reduction of displaced distal radius fracture?

**DOI:** 10.1186/s13018-018-0772-7

**Published:** 2018-03-27

**Authors:** Ping-Tao Tseng, Tsai-Hsueh Leu, Yen-Wen Chen, Yu-Pin Chen

**Affiliations:** 1 Department of Orthopaedic Surgery, Wan Fang Hospital, School of Medicine, College of Medicine, Taipei Medical University, Number 111, Section 3, Xinglong Road, Wenshan District, Taipei City, 116 Taiwan; 2 WinShine Clinics in Specialty of Psychiatry, Kaohsiung City, Taiwan; 3 Prospect clinic for otorhinolaryngology & neurology, Kaohsiung City, Taiwan

**Keywords:** Hematoma block, Procedural sedation and analgesia, Radius fracture

## Abstract

**Background:**

Procedure sedation and analgesia (PSA) is often used to alleviate discomfort and to facilitate fracture reduction for patients with distal radius fracture in emergency departments and clinics, but risks of respiratory distress and needs for different levels of monitoring under PSA are still under concern. Hematoma block (HB) is a simple alternative method of providing rapid pain relief during reduction of distal radius fracture. However, there is still in lack of strong evidence to promote HB over PSA in clinical practice. The aim of this study was to compare HB and PSA for adult and pediatric patients during reduction of displaced distal radius fracture to identify the level of pain relief, frequency of adverse effects (AEs), and reduction failure.

**Methods:**

The PubMed, ScienceDirect, Cochrane Library, and ClinicalTrials.gov were searched for studies comparing HB or PSA in distal radius fracture reduction. The search revealed four randomized controlled trials and one non-randomized trial, which included two studies of pediatric subjects and three studies of adult subjects. Subgroup meta-analysis for adult and pediatric groups were specifically performed according to age difference to avoid potential bias.

**Results:**

In the adult group, the effect of HB on post-reduction pain severity was better than that of PSA with significant heterogeneity (Hedges’ *g* − 0.600, 95% confidence interval (CI) − 1.170 to − 0.029, *p* = 0.039), although there was no difference on the pain severity during reduction between these two groups with significant heterogeneity (Hedges’ *g* 0.356, 95% CI − 1.101 to 1.812, *p* = 0.632). In the pediatric group, the treatment effect on pain severity was significantly better by HB than that by PSA but without significant heterogeneity (Hedges’ *g* − 0.402, 95% CI − 0.718 to − 0.085, *p* = 0.013, *I*^*2*^ < 0.001%). Most of the reported adverse effects (AEs) include nausea, vomiting, and respiratory distress developed in adult patients treated by PSA. The rates of reported AEs did not significantly differ between HB and PSA in the pediatric group. Additionally, final outcomes of reduction failure did not significantly differ between HB and PSA in both adult and pediatric groups.

**Conclusion:**

Hematoma block is a safe and effective alternative of anesthesia in reduction of distal radius fracture without inferior pain relief compared with PSA among adult and pediatric patients.

**Electronic supplementary material:**

The online version of this article (10.1186/s13018-018-0772-7) contains supplementary material, which is available to authorized users.

## Background

Distal radius fractures are the most common fracture of the upper extremity with bimodal peak incidence in both the pediatric and elderly population [1]. With improved understanding of biomechanics as well as clinical research, reduction of the fracture displacement has been generally accepted as the initial management to reduce local tissue pressure and release of discomforts, either in situation further open reduction and internal fixation is needed or not [2]. For pediatric population, exact and delicate manual reduction is by no means important, since closed reduction and casting of these fractures can provide definitive treatment [3]. However, painful feelings during manual reduction can not only cause discomforts and stress to the patients but also interfere with successful reduction.

Procedural sedation and analgesia (PSA), defined as a technique of administering sedatives or dissociative agents with or without analgesics to induce a state that allows the patient to tolerate unpleasant procedures while maintaining cardiorespiratory function, is commonly utilized to reduce patient discomfort during manual reduction of displaced distal radius fracture in the emergency department outside the operating room [4, 5]. Pharmacologic options for PSA include a short-acting benzodiazepine, either alone or in combination with an opioid analgesic [6]. Evidence to support the use of other sedatives including etomidate and propofol for PSA is also emerging in the literature [7] However, PSA has its own risks and considerations for different levels of monitoring for cardiorespiratory function. Hematoma block (HB), defined as a procedure with local anesthetic injected directly into the fracture site, is a safe and effective alternative technique for pain control in assistance with manual reduction for distal radius fracture [8]. Its potential benefits include avoidance of PSA-associated risks, high cost-effectiveness, and time-sparing procedure. However, the highest level-evidence assessment relying on results from meta-analyses in 2002 cannot demonstrate the relative effectiveness of different methods of anesthesia including HB and PSA owing to lack of enough evidence from randomized trials [9].

In recent years, evidence from well-conducted randomized controlled trials has confirmed the effectiveness of HB in assistance with manual reduction of distal radius in both the adult and pediatric patients [10–12]. But, as of this time, we are not aware of any update meta-analyses evaluating the overall benefits and harms between HB and PSA in closed reduction of distal radius fracture. Therefore, the purpose of this meta-analysis was to give an evaluation of the updated clinical evidence from randomized trials and well-controlled intervention studies.

## Method

The current meta-analysis was conducted according to the *Preferred Reporting Items for Systematic Reviews and Meta-Analyses* (PRISMA) statement [13] (Additional file 1: Table S1), which had been widely used as guideline of meta-analysis of interventional trials.

### Inclusion and exclusion criteria

The inclusion criteria were the following: (1) published randomized controlled trials (RCTs) comparing the efficacy of HB and PSA during reduction of distal radius fracture on pain control, (2) articles including patients with the diagnosis of distal radius fracture, and (3) articles that were clinical trials including adult or pediatric populations. No limit was set for the length of study follow-up. The exclusion criteria were (1) articles that were not controlled intervention studies, (2) animal studies, and (3) articles comparing the efficacy of HB and PSA after reduction or operation.

### Database searches and identification of eligible papers

Two authors (YP Chen and PT Tseng) searched PubMed, ScienceDirect, Cochrane Library, and ClinicalTrials.gov using the keywords of “(radius fracture) AND (hematoma block OR local anesthesia)” from inception to July 28, 2017 without any limitations applied. In addition, the reference lists of review articles relevant to this topic were manually searched to identify potentially eligible papers [9, 14].

After completing the searches, duplicate studies were removed, and the above two authors independently screened the titles and abstracts for eligibility. A list of potentially eligible studies was developed by consensus, and the full texts were assessed by the two authors. Both authors then applied the eligibility criteria and developed a final list of studies to be included. A third reviewer was available for mediation in the event of any inconsistencies.

### Methodological quality appraisal

The methodological quality of the included studies was determined by two authors using the Jadad scale [15]. In brief, the Jadad score was calculated for each study and included three categories of study quality: randomization, blindness, and withdrawals and dropouts. The Jadad score ranged from zero (poor quality) to five (high quality).

### Primary outcomes

The primary outcomes were the differences in pain obtained by HB and PSA during reduction procedures. The outcomes of interest were recorded using validated scales with specific scoring systems, e.g., visual analogue scale (VAS) [16], numeric pain rating scale (NRS) [17], Wong-Baker FACES Pain Rating Scale [18], or Procedure Behavior Checklist (PBCL) [19].

### Secondary outcomes

The secondary outcomes of interest included differences in pain severity after reduction procedure by HB or PSA, differences in reduction failure, and adverse effects (AEs) between HB or PSA during reduction.

### Data extraction and management

The two authors extracted data from the eligible studies into a database. The variables of interest included mean age (years), gender, pain severity, ethnicity (Caucasian, African-American, Hispanic, Asian, or Native American), and Jadad score. For articles that did not provide data, we contacted the corresponding authors twice over a month-long period in an attempt to acquire the variables of interest.

### Meta-analysis

Under the presumed heterogeneity of the sample populations among all of the recruited studies, the data were analyzed using Comprehensive Meta-Analysis software, version 3 (Biostat, Englewood, NJ). The analytical models were random-effects meta-analysis models rather than fixed effect models [20]. Since not all studies used the same metrics to estimate the same target (pain severity), we converted and merged all the effect sizes (ESs) of different scales regarding pain severity with the Hedges’ *g* and 95% confidence intervals (95% CIs) according to the protocol in the Comprehensive Meta-Analysis manuals and guidance on the Comprehensive Meta-Analysis website (https://www.meta-analysis.com/downloads/Meta-analysis%20Converting%20among%20effect%20sizes.pdf). A two-tailed *p* value less than 0.05 was considered statistically significant.

### Heterogeneity, publication bias, and sensitivity test

Heterogeneity was assessed using the Q statistic to evaluate the dispersion of the true effect among the recruited studies [21]. The *I*^*2*^ statistic was used to indicate the proportion of heterogeneity among study estimates [22]. Publication bias was evaluated by visually inspecting funnel plots [23] and by performing Egger’s regression tests [24]. In cases of publication bias, the Duval and Tweedie trim and fill test was performed to impute potential missing trials and re-calculate the effect size [25]. Sensitivity was tested by performing one-study removal to examine whether the results of our meta-analysis were influenced by any outliers within the recruited studies [26].

### Meta-regression and subgroup meta-analysis

Finally, meta-regression and subgroup meta-analyses were performed to investigate the effects of clinical variables as possible sources of heterogeneity. Meta-regression analyses were performed using the unrestricted maximum likelihood method only when data were available across more than five studies. Subgroup meta-analysis was performed when at least two datasets were available.

## Results

### Study selection

**Fig. 1:**
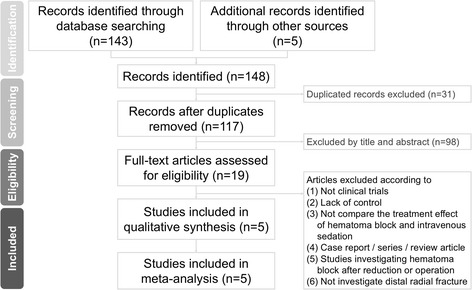
Flowchart of the current meta-analysis. The databases used, the inclusion criteria, the search strategy, and selection process of the current meta-analysis

After screening the titles/abstracts, nineteen full-text articles were assessed for eligibility. Of these, fourteen were excluded (Fig. 1 and Additional file 2: Table S2). Therefore, this meta-analysis included five articles [10–12, 27, 28]. Of these, four were randomized controlled trials [10, 12, 27, 28], and one was a non-randomized controlled trial [11] in which subjects were subdivided according to patients and family’s own will. Pain severity was evaluated by PBCL in one study [27], by Wong-Baker FACES in one study [11], by NRS pain score in one study [10], and by VAS in two studies [12, 28]. Furthermore, the subjects were adolescents in two studies (children with HB = 73, mean age = 10.3, mean female proportion = 32.7; children with PSA = 81, mean age = 10.3, mean female proportion = 34.7) [11, 27], and the subjects were adults in the other three studies (patients with HB = 152, mean age = 43.9, mean female proportion = 48.0; patients with PSA = 153, mean age = 43.4, mean female proportion = 47.7) [10, 12, 28]. To avoid potential bias, these two distinct groups were not pooled to perform meta-analysis. To be specific, subgroup meta-analysis was performed according to age difference.

### Methodological quality of the included studies

Across the five studies, the average Jadad score was 2.80 with a standard deviation (SD) of 1.48 (Additional file 3: Table S3).

### Meta-analyses that compared HB and PSA for treating pain in adult patients

#### Pain during reduction procedure

**Fig. 2:**
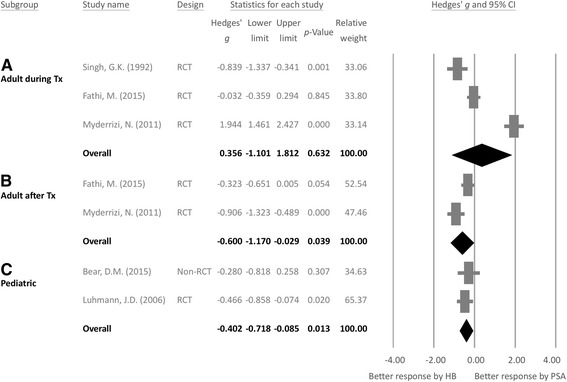
Forest plot of the difference in pain severity between hematoma block and intravenous sedation (A) during reduction procedure in adult patients, (B) after reduction procedure in adult patients, and (C) in pediatric patients. (A) shows that pain did not significantly differ during reduction procedure between HB and IVS in adult patients (Hedges’ *g* 0.356, 95% CI − 1.101 to 1.812, *p* = 0.632); (B) the treatment effect on pain severity was significantly better in adult patients who received HB compared to those who received PSA (Hedges’ *g* − 0.600, 95% CI − 1.170 to − 0.029, *p* = 0.039); and (C) the treatment effect on pain severity was significantly better by HB than that by PSA in pediatric patients (Hedges’ *g* − 0.402, 95% CI − 0.718 to − 0.085, *p* = 0.013)

The three studies of adult patients provided three datasets about the difference of pain during reduction procedure between HB and PSA [10, 12, 28]. The meta-analysis results showed that HB and PSA did not significantly differ in pain reduction in adult patients (Hedges’ *g* 0.356, 95% CI − 1.101 to 1.812, *p* = 0.632) (Fig. 2(A)) with significant heterogeneity (*Q* value = 68.544, df = 2, *p* < 0.001; *I*^*2*^ = 97.082%, tau = 1.267) but no publication bias according to inspection of funnel plot (Additional file 4: Figure S1) or Egger’s test (*t* = 0.260, df = 1, 2-tailed *p* = 0.838).

#### Sensitivity test

The sensitivity test showed that non-significant differences did not change after removal of any one of the recruited studies. Therefore, non-significant differences did not result from outliers.

#### Meta-regression and subgroup meta-analysis

Further meta-regression or subgroup meta-analyses were not performed because not enough trials were available.

#### Pain after reduction procedure

Of the three studies of adults, only two provided total datasets about the difference in pain after reduction procedure between HB and PSA. Figure 2b shows the meta-analysis results for adult patients, which revealed that HB obtained a significantly larger reduction in pain compared to PSA (Hedges’ *g* − 0.600, 95% CI − 1.170 to − 0.029, *p* = 0.039) with significant heterogeneity (*Q* value = 4.631, df = 1, *p* = 0.031; *I*^*2*^ = 78.406%, tau = 0.365). Publication bias was not evaluated because less than three datasets were used.

#### Sensitivity test

The sensitivity test revealed that the results of meta-analysis would change into insignificance after removal of the datasets by Myderrizi and Mema (Hedges’ *g* − 0.323, 95% CI − 0.651 to 0.005, *p* = 0.054) [12], which might be due to much less sample size after removal of that study than before.

#### Meta-regression and subgroup meta-analysis

Further meta-regression or subgroup meta-analysis was not performed because not enough trials were available.

### Meta-analysis investigating the different effect of pain treatment by HB and PSA in pediatric patients

The meta-analysis results further revealed that, in pediatric patients, HB also obtained a larger reduction in pain severity compared to PSA (Hedges’ *g* − 0.402, 95% CI − 0.718 to − 0.085, *p* = 0.013) (Fig. 2(C)) without significant heterogeneity (*Q* value = 0.300, df = 1, *p* = 0.584; *I*^*2*^ < 0.001%, tau < 0.001). Publication bias was not evaluated because less than three datasets were used.

#### Sensitivity test

The sensitivity test revealed that the results of meta-analysis would change into insignificance after removal of the datasets by Luhmann (Hedges’ *g* − 0.280, 95% CI − 0.818 to 0.258, *p* = 0.307) [27], which might be due to much less sample size after removal of that study than before.

#### Meta-regression and subgroup meta-analysis

Further meta-regression or subgroup meta-analysis was not performed because not enough trials were available.

### Secondary outcome

Further meta-analyses of secondary outcome, including differences of reduction failure after reduction procedure as well as differences in complications and AEs, were not compared between HB or PSA because less than two datasets were available.

Additional file 5: Table S4 summarizes the secondary outcomes. In the adult patient group, Myderrizi et al. and Singh et al. reported no difference in the rate of reduction failure at the early or late follow-up respectively [12, 28]. In the pediatric patient group, Bear et al. also reported no difference in the rate of further reduction at the final follow-up [11]. Furthermore, nausea, vomiting and respiratory distress were the most common AEs developing after HB or PSA. In the adult patients analyzed by Fathi et al., 11% had early AEs in the PSA group, but no patients had AEs in the HB group [10]. In the pediatric group, Bear et al. reported 7.7% self-limited paresthesia after HB [11]. However, the rates of other AEs did not significantly differ between HB and PSA in Bear et al. and Luhmann et al. [11, 27].

## Discussion

In this context, we performed a meta-analysis comparing the most common two methods of anesthesia, PSA and HB, used in emergency departments and clinics for reduction of distal radius fracture to compare level of pain relief, the risk of treatment failure as well as the frequency of complications. This meta-analysis showed that HB is a safe and effective alternative to PSA for facilitating pain relief during distal radius fracture reduction in adult and pediatric patients. Specifically, HB obtained a significantly larger reduction in pain severity compared to PSA in adult patients after reduction of fracture and for pediatric population during reduction of fracture.

In addition to HB and PSA, several methods of anesthesia have also been advocated for alleviating discomfort and facilitating reduction in patients with displaced distal radius fracture, including intravenous regional anesthesia (Bier block) and regional nerve blocks [9, 29, 30]. Intravenous regional anesthesia (Bier block) has proven effective for anesthesia during upper limb surgery [31, 32]. However, concerns still exist over the complications including pain at the tourniquet site, local anesthetic toxicity, and instant recurrence of pain at surgical site following tourniquet deflation and the serious dangers of leakage of anesthetic after accidental deflation of the tourniquet [33, 34]. Regional nerve block is another effective method of controlling pain during fracture reduction [35]. Nevertheless, highly skill-dependent technique may prevent the clinicians from general application of regional nerve block when treating distal radius fracture.

The PSA is widely used in the emergency department as a part of daily practice in both university and community hospitals, but patients undergoing PSA may be under risks of respiratory distress and need different levels of monitoring. The HB provides a simple and alternative technique for pain relief during reduction of distal radius fracture. This meta-analysis showed that, in adult patients, for relief of larger post-reduction pain in adult patients, HB is superior to PSA. However, there was no difference on the pain severity during reduction between these two groups. This implies that anesthesia with HB, instead of PSA, is beneficial for maintaining the effect of pain relief after fracture reduction. In the pediatric patients, treatment by HB also revealed less pain severity compared with that by PSA. Additionally, the adult patients treated by PSA suffered most AEs, including nausea, vomiting, and respiratory distress. The rates of reported AEs did not differ between pediatric patients receiving HB and those receiving PSA, nor difference on the final outcomes of reduction failure between groups in adult and pediatric patients.

### Limitations

This meta-analysis has several limitations. First, although only RCTs and well-controlled intervention studies were considered for inclusion, study quality varied among the recruited studies. Potential sources of bias in these trials included inadequate methods to conceal random allocation as well as lack of blinding. Second, the intent was to compare the benefits and effectiveness between HB and PSA. However, the regimens of intervention varied among these enrolled studies [10–12, 27, 28]. For example, options of local anesthesia for HB in our enrolled studies included lidocaine and xylocaine of varying concentrations. In addition, the PSA regimens also widely varied among adult and pediatric patients, which may also contribute to variable AEs and result in heterogeneity and potential bias. However, in clinical practice, it was unavoidable to introduce additional sedation procedure to avoid irritation or agitation during reduction of distal radius fracture when HB was performed in pediatric patients. This additional sedation procedure with oral midazolam or inhalant nitrous oxide had been seen in the current study and may also increase events of AEs [11, 27]. To avoid this potential bias, adult and pediatric groups were separated to perform subgroup meta-analysis according to adult/pediatric age difference. However, since subgroup analysis dilutes the number of datasets and cases, it would have decreased the power of the conclusion achieved by our meta-analysis. Third, the technique used to perform HB in the adult population also varied. For example, in Fathi et al., HB was guided by ultrasound to augment the efficacy of pain relief owing to better and more accurate local injection of anesthetics into the fracture site so as to potentially enhance effect of pain relief [10]. Last but not least, quality of fracture reduction plays an important role in successful treatment of displaced distal radius fracture and may decide the risk of reduction loss. Besides, cost-effectiveness is also another important point of view for comparing the outcome between HB and PSA during reduction of displaced distal radius fracture. However, the pity of it is that all of the enrolled studies in our meta-analysis focus neither on the quality of fracture reduction nor on the cost of the different methods of anesthesia. More well-organized randomized control trials, including considerations for outcomes of reduction quality and cost-effectiveness under HB or PSA, may be necessary for future research in this field.

## Conclusion

This meta-analysis showed that HB is a safe and effective alternative method of anesthesia in reduction of distal radius fracture without inferior pain relief compared with PSA among adult and pediatric patients. However, owing to insufficient robust evidence from a large number of datasets, future RCTs should include more restrictions to limit bias, including concealed randomization, larger sample sizes, objective outcome measures, and blinded outcome assessments.

## Additional files

Additional file 1: **Table S1.** PRISMA checklist of current meta-analysis. (DOCX 27 kb)

Additional file 2: **Table S2.** Excluded studies and reason. (DOCX 17 kb)

Additional file 3: **Table S3.** Jadad scores of recruited studies. (DOCX 16 kb)

Additional file 4: **Figure S1.** Funnel plot of meta-analysis of different pain during reduction procedure. (PNG 91 kb)

Additional file 5: **Table S4.** Summary of secondary outcome among the reference. (DOCX 20 kb)

## Abbreviations

CI: Confidence interval
HB: Hematoma block
non-RCT: Non-randomized controlled trial
PSA: Procedural sedation and anesthesia
RCT: Randomized controlled trial
Tx: Treatment
