# Supplementary material for: Hematoma block or procedural sedation and analgesia, which is the most effective method of anesthesia in reduction of displaced distal radius fracture?
Source: J Orthop Surg Res. 2018 Mar 27;13:62. doi: 10.1186/s13018-018-0772-7 (PMC5869786; doi:10.1186/s13018-018-0772-7)
Supplement: Supplementary file 2 — Table S2. Excluded studies and reason. (DOCX 17 kb) [file 13018_2018_772_MOESM2_ESM.docx]

**Additional file 2: Table S2:** Excluded studies and reason

Not compare the treatment effect of hematoma block and intravenous sedation (n=7)

Graham CA, Gibson AJ, Goutcher CM, et al. Anaesthesia for the management of distal radius fractures in adults in Scottish hospitals. *Eur J Emerg Med* 1997;4(4):210-2.

Zamzam MM, Khoshhal KI. Displaced fracture of the distal radius in children: factors responsible for redisplacement after closed reduction. *J Bone Joint Surg Br* 2005;87(6):841-3. doi: 10.1302/0301-620X.87B6.15648

Ogunlade SO, Omololu AB, Alonge TO, et al. Haematoma block in reduction of distal radial fractures. *West Afr J Med* 2002;21(4):282-5.

Edmonds EW, Capelo RM, Stearns P, et al. Predicting initial treatment failure of fiberglass casts in pediatric distal radius fractures: utility of the second metacarpal-radius angle. *J Child Orthop* 2009;3(5):375-81. doi: 10.1007/s11832-009-0198-1

Constantine E, Steele DW, Eberson C, et al. The use of local anesthetic techniques for closed forearm fracture reduction in children: a survey of academic pediatric emergency departments. *Pediatr Emerg Care* 2007;23(4):209-11. doi: 10.1097/PEC.0b013e31803e1792

Haasio J. Cubital nerve block vs haematoma block for the manipulation of Colles' fracture. *Ann Chir Gynaecol* 1990;79(3):168-71.

Kendall JM, Allen P, Younge P, et al. Haematoma block or Bier's block for Colles' fracture reduction in the accident and emergency department--which is best? *J Accid Emerg Med* 1997;14(6):352-6.

Review article (n=1)

Handoll HH, Madhok R. Closed reduction methods for treating distal radial fractures in adults. *The Cochrane database of systematic reviews* 2003(1):CD003763. doi: 10.1002/14651858.CD003763

Arranged hematoma block after operation but not before/during procedure (n=2)

Chung MS, Roh YH, Baek GH, et al. Evaluation of early postoperative pain and the effectiveness of perifracture site injections following volar plating for distal radius fractures. *J Hand Surg Am* 2010;35(11):1787-94. doi: 10.1016/j.jhsa.2010.07.023

Peel WJ, McNicholas M, Colvin JR, et al. Haematoma block: Assessment of postoperative analgesic effect following manipulation of distal forearm fractures. *Acute Pain* 1999;2(1):33-35.

Case report (n=1)

Gottlieb M, Cosby K. Ultrasound-guided hematoma block for distal radial and ulnar fractures. *J Emerg Med* 2015;48(3):310-2. doi: 10.1016/j.jemermed.2014.09.063

Lack of control (n=1)

Jimenez A, Blazquez D, Cruz J, et al. Use of combined transmucosal fentanyl, nitrous oxide, and hematoma block for fracture reduction in a pediatric emergency department. *Pediatr Emerg Care* 2012;28(7):676-9. doi: 10.1097/PEC.0b013e31825d20f6

Compared radiology change but not pain severity (n=1)

Hoffmann M, Schroeder M, Kossow K, et al. Radiological dorsal tilt analysis of AO type A, B, and C fractures of the distal radius treated conservatively or with extra-focal K-wire plus external fixateur. *Skeletal Radiol* 2012;41(9):1133-9. doi: 10.1007/s00256-011-1355-4

Compared radial head fractures but not distal radius fracture (n=1)

Chalidis BE, Papadopoulos PP, Sachinis NC, et al. Aspiration alone versus aspiration and bupivacaine injection in the treatment of undisplaced radial head fractures: a prospective randomized study. *J Shoulder Elbow Surg* 2009;18(5):676-9. doi: 10.1016/j.jse.2009.04.003
