# Supplementary material for: Hematoma block or procedural sedation and analgesia, which is the most effective method of anesthesia in reduction of displaced distal radius fracture?
Source: J Orthop Surg Res. 2018 Mar 27;13:62. doi: 10.1186/s13018-018-0772-7 (PMC5869786; doi:10.1186/s13018-018-0772-7)
Supplement: Supplementary file 3 — Table S3. Jadad scores of recruited studies. (DOCX 16 kb) [file 13018_2018_772_MOESM3_ESM.docx]

**Additional file 3: Table S3:** Jadad scores of recruited studies

| Author (year) | Country | Jadad score | | | |
| --- | --- | --- | --- | --- | --- |
|  |  | Randomization | Blindness | Cohort | Total |
| Bear, D.M. (2015) | USA | 0 | 0 | 1 | 1 |
| Fathi, M. (2015) | Iran | 2 | 0 | 1 | 3 |
| Myderrizi, N. (2011) | Albania | 1 | 0 | 1 | 2 |
| Luhmann, J.D. (2006) | USA | 2 | 0 | 1 | 3 |
| Singh, G.K. (1992) | India | 2 | 2 | 1 | 5 |
