# Supplementary figures and images for: Hematoma block or procedural sedation and analgesia, which is the most effective method of anesthesia in reduction of displaced distal radius fracture?
Source: J Orthop Surg Res. 2018 Mar 27;13:62. doi: 10.1186/s13018-018-0772-7 (PMC5869786; doi:10.1186/s13018-018-0772-7)

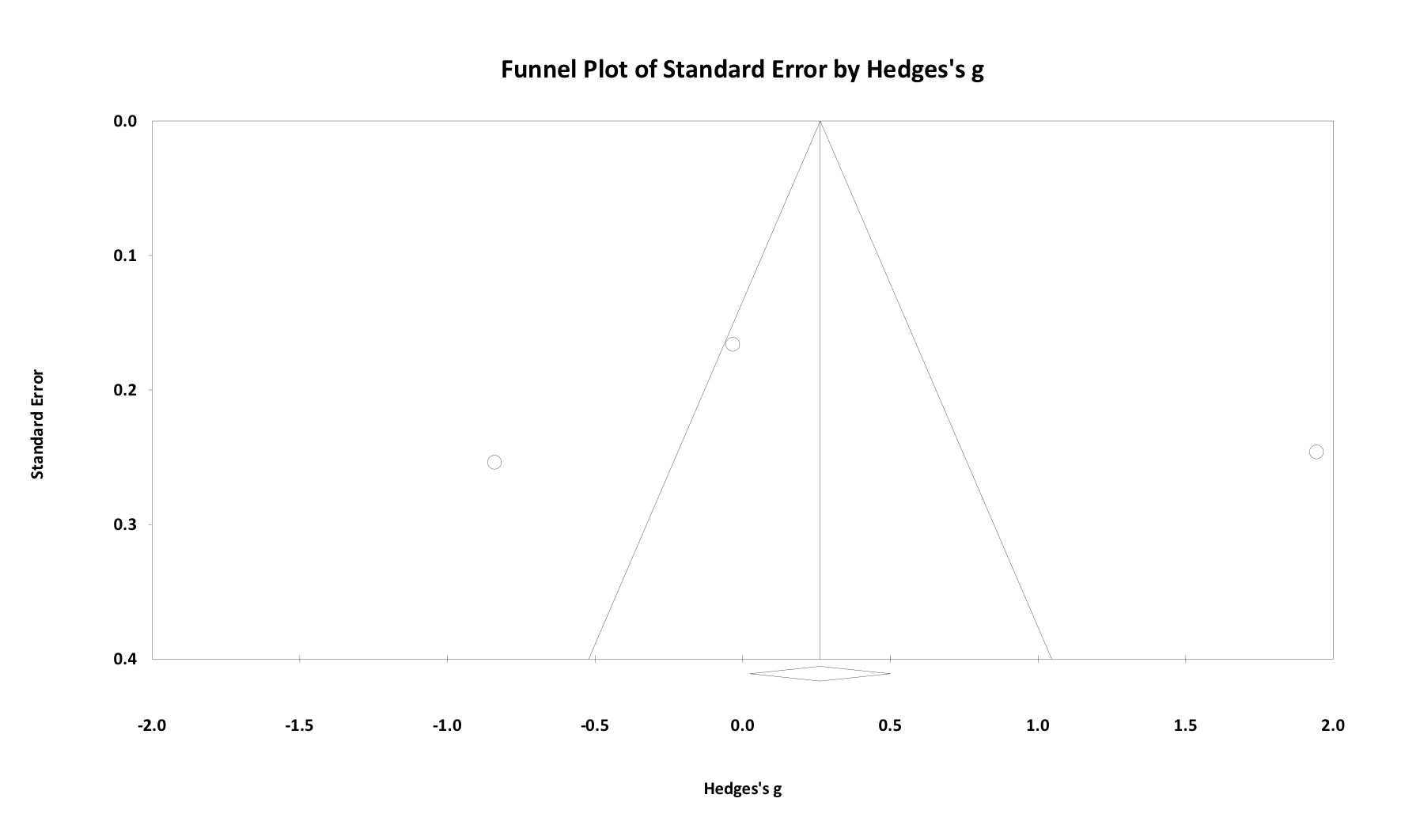

Supplement: Supplementary file 4 — Figure S1. Funnel plot of meta-analysis of different pain during reduction procedure. (PNG 91 kb) [file 13018_2018_772_MOESM4_ESM.png]
