# Supplementary material for: Hematoma block or procedural sedation and analgesia, which is the most effective method of anesthesia in reduction of displaced distal radius fracture?
Source: J Orthop Surg Res. 2018 Mar 27;13:62. doi: 10.1186/s13018-018-0772-7 (PMC5869786; doi:10.1186/s13018-018-0772-7)
Supplement: Supplementary file 5 — Table S4. Summary of secondary outcome among the reference. (DOCX 20 kb) [file 13018_2018_772_MOESM5_ESM.docx]

| **Reference** | **Intervention** | **Secondary outcome** | |
| --- | --- | --- | --- |
|  |  | **Reduction failure** | **Adverse effect (AE)** |
| **Adult population** | | | |
| Fathi, M. (2015) | Ultrasound-guided HB  versus PSA | No report | 11% (8/72) early AE (1 vomiting, 3 apnea, 2 hypotension, 2 respiratory depression), no late AE in PSA group; no early and late AE in HB |
| Myderrizi, N. (2011) | HB versus PSA | Loss of reduction on the 1st week after reduction:  43% (21/48) for HB; 45% (22/48) for PSA group | No report |
| Singh, G.K. (1992) | HB versus PSA | Failure on the post-reduction 8th week:  6% (2/33) for HB; 12% (4/33) for PSA group | No report |
| **Pediatric population** | | | |
| Bear, D.M. (2015) | HB + oral midazolam  Versus PSA | One patient in the HB group and one patient in the PSA group needed revision closed reduction and casting; One in PSA group needed closed reduction and percutaneous pinning | 3.8% (1/26) for PSA group and 3.8% (1/26) for HB group with nausea; 7.7% (2/26) for HB group with self-limited paresthesia |
| Luhmann, J.D. (2006) | HB + inhaled N_2_O  versus PSA | No report | PSA group versus HB/N2O group: 24% versus 26% with vomiting; 7% versus 0% with difficulty breathing; 47% versus 44% with post discharge lethargy |

**Additional file 5: Table S4:** Summary of secondary outcome among the reference

Abbreviation: HB: hematoma block; PSA: procedural sedation and anesthesia; AE: adverse effect
